# Anomaly Behavior Detection Based on Deep Learning in an IoT Environment

**DOI:** 10.3390/s25247605

**Published:** 2025-12-15

**Authors:** Anqi Fu, Jian Li

**Affiliations:** School of Cyberspace Security, Beijing University of Posts and Telecommunications, Beijing 100876, China

**Keywords:** intelligent sensing, video anomaly detection, temporal structural attention, contrastive learning

## Abstract

In the era of the Internet of Things (IoT), video surveillance, as a vital component of smart cities and public security systems, faces the critical challenge of efficiently detecting abnormal behaviors within massive video streams. However, existing weakly supervised video anomaly detection methods are often limited by the scarcity of abnormal samples, the similarity between normal and abnormal segments, and the insufficient modeling of temporal dependencies. To address these challenges, this paper proposes a novel approach that integrates temporal structural attention with contrastive learning. On the one hand, causal masks and temporal decay weights are incorporated into the attention mechanism to explicitly constrain temporal relations and prevent future information leakage; on the other hand, positive/negative offsets and a contrastive learning strategy are employed to enhance the discriminability of abnormal segments in the latent space. Experiments conducted on multiple public video anomaly detection datasets validate the effectiveness of the proposed method, with results showing superior performance over existing mainstream models: the AUC increases to 98.1%, ACC reaches 96.1%, and the F1-score improves to 94.5%. These findings demonstrate that the proposed method can provide more intelligent, efficient, and reliable anomaly detection for IoT-based video surveillance, holding significant implications for public safety and intelligent monitoring.

## 1. Introduction

With the rapid advancement of the Internet of Things (IoT) and intelligent surveillance, video surveillance systems have been widely deployed in various domains, including public safety, traffic management, and social governance. How to automatically and accurately identify potential anomalous events within massive video data streams has become a critical issue in smart cities and public safety systems. In this context, Video Anomaly Detection (VAD) is a key task in intelligent perception and edge computing, which can assist systems in real-time detection of potential risk events in complex environments, providing strong support for building efficient and intelligent security and prevention systems. In this study, the IoT environment primarily refers to intelligent video surveillance scenarios. The datasets used in our experiments, such as ShanghaiTech and UCF-Crime, consist of video clips captured from real surveillance systems, which align with typical IoT-based monitoring applications. Compared to traditional methods that rely on manual inspections, deep learning-based automated anomaly detection not only significantly reduces labor costs but also improves response speed and the intelligence level of monitoring systems.

Despite the emergence of numerous deep learning-based research results in recent years, video anomaly detection still faces a series of challenges. First, anomalous events are typically rare and abrupt, making frame-level annotation extremely costly. Consequently, most existing studies adopt a weakly supervised learning framework that relies solely on video-level labels [1]. However, the very low proportion of anomalous segments within videos, coupled with the high similarity between their feature distribution and that of normal segments, often leads to model confusion and consequently, a decline in detection performance. Second, insufficient modeling of temporal dependencies presents another major bottleneck. Current mainstream attention mechanisms primarily capture static correlations between segments but often neglect causality and temporal dynamics. This limitation hinders the model’s ability to effectively capture the feature differences in anomalous behaviors across time series [2,3]. In addition, existing methods are sensitive to noise and have poor generalization ability, which limits their application in real IoT scenarios. In summary, the key problem addressed in this paper is how to reduce the confusion caused by anomaly sparsity and feature similarity, while at the same time improving the modeling of temporal dynamics under weakly supervised conditions.

In response to the aforementioned challenges, researchers have explored various improved approaches. For example, a temporal analysis method based on Gray Level Co-occurrence Matrix (GLCM) [4] features has been used to detect violent and abnormal crowd activities. This method models crowd dynamics by encoding the changes in crowd texture and introduces an inter-frame uniformity measure (IFU) to describe the emergence of violent behaviors. However, its performance is limited in complex scenarios, primarily because it is difficult to accurately capture individual behaviors in dense crowds or under heavy occlusion, thereby adversely affecting detection performance. Additionally, a video prediction model based on Long Short-Term Memory (LSTM) networks [5] has been proposed to capture the spatiotemporal dependencies in video sequences. By incorporating causal LSTM units and gradient highway structures, the aim is to mitigate the vanishing gradient problem. Although this approach enhances the modeling of long-range dependencies to some extent, the model still struggles with issues like gradient vanishing and capturing sufficiently long-range dependencies, particularly when handling anomalous behaviors in complex scenes, where its performance remains limited. Graph Convolutional Networks (GCN) have been introduced to capture the graph-structured relationships between segments [6], showing certain advantages in modeling high-dimensional temporal features. Memory-based methods [7] explicitly store prototypical normal patterns to enhance the discrimination of anomalies, but they can be vulnerable to limitations in memory capacity and interference from noisy data. However, these methods still face issues such as insufficient temporal dynamic modeling and inadequate separation of anomalous segments under weakly supervised conditions.

To address these limitations, this paper proposes a weakly supervised video anomaly detection method that integrates a temporal structural attention mechanism with contrastive learning. Specifically, the motivation for the contrastive learning module is to address the challenge of feature similarity between normal and anomalous segments. By introducing a lightweight one-dimensional convolutional predictor and optimizing the InfoNCE loss, the model explicitly separates anomalous and normal segments in the latent space, thereby sharpening their decision boundaries. The motivation for the temporal structural attention mechanism is to remedy the inadequate modeling of temporal dynamics in prior methods. By employing causal masks to prevent information leakage from the future and incorporating temporal decay functions to model long-range dependencies with attenuation, the module captures both causality and temporal evolution of anomalies. These two components work synergistically: contrastive learning enhances the discriminability of features, while temporal structural attention improves the sensitivity to temporal variations in anomalous events. Together, they form a logically consistent framework that directly responds to the weaknesses of prior methods and effectively alleviates the challenges of anomaly sparsity and feature similarity under weak supervision. Experimental results show that the proposed method significantly outperforms existing mainstream methods on multiple benchmark datasets, validating the effectiveness of the approach.

The main contributions of this paper can be summarized as follows:

(1) To address the issue of insufficient temporal modeling in weakly supervised video anomaly detection, a temporal structural attention mechanism is proposed, which incorporates causal masks and temporal decay weights, effectively enhancing the ability to model temporal dependencies;
(2) A contrastive learning module based on positive and negative shifts is designed to enhance the separation between anomalous and normal segments in the latent space;
(3) Experimental validation is conducted on multiple benchmark datasets, with results showing that the proposed method outperforms existing mainstream models in terms of both accuracy and robustness, demonstrating promising application prospects.

In summary, by combining temporal structural attention and contrastive learning, this paper provides an efficient and robust solution for video surveillance in IoT scenarios. It holds significant implications for the intelligent development of smart cities and public safety systems.

## 2. Related Work

### 2.1. Research Status of Abnormal Behavior Recognition Based on Deep Learning

In the field of computer vision, abnormal behavior recognition is a crucial technology, with significant implications for various application scenarios such as intelligent surveillance, human–computer interaction, and autonomous driving. With the rapid advancement of deep learning technology, deep learning-based human behavior recognition methods have gradually replaced traditional handcrafted feature extraction techniques, becoming the mainstream approach in this domain. Zhan Chuqing et al. [8] proposed a human behavior recognition algorithm based on deformable 3D convolution and Bert temporal modeling. By integrating deformable convolution with 3D convolutional neural networks (CNNs) and combining it with Bert temporal modeling, they significantly improved the model’s accuracy and reduced the loss function value on the HMDB51 dataset. Experimental results showed that this algorithm achieved notable success in enhancing model performance. Zhang Han and Feng Jiahong [9] systematically reviewed the application of deep learning in human behavior recognition, highlighting that deep learning methods, with their powerful automatic feature learning capabilities, effectively address the limitations of traditional methods, such as poor generalization of handcrafted features and insufficient adaptability to complex scenarios. Gong Yiwen et al. [10] used a greedy algorithm to extract human key points from video frames and combined affinity comparison rules to obtain human skeleton feature parameters. These parameters were then input into the Inception v3 deep learning model for training, resulting in a pedestrian abnormal behavior detection model. Their experimental results demonstrated that deep learning-based pedestrian abnormal behavior recognition achieved higher accuracy and faster computation speed.

In terms of method evolution, Guo Jianjun et al. [11] argued that traditional human behavior recognition, which relied on handcrafted feature extraction, although foundational for early research, gradually became inadequate due to weak feature generalization and insufficient adaptability to complex environments. Deep learning-based methods, however, overcame these limitations by automatically learning features through the network. Research in this field can be broadly categorized into several core directions, with CNNs serving as a key foundation. Lin Feng [12] proposed a CNN-based recognition method for human behavior classification using smartphone triaxial accelerometer data. This method segmented continuous sample data into fixed time windows, constructed a multi-layer neural network structure, and optimized core parameters. The final trained model achieved an average cross-validation accuracy of 91.7%, significantly outperforming traditional machine learning algorithms and providing an efficient solution for behavior recognition using sensor data.

Md. Alamin Talukder et al. [13] developed a composite framework integrating advanced architectures such as CvLSTM, LRCN, and BiLSTM, which combine spatial feature extraction with time-series modeling capabilities. In dataset testing, the BiLSTM component, with its bidirectional temporal awareness advantage, achieved high precision recognition rates of 98.67% and 94.23%, surpassing single architectures across multiple metrics, including precision and recall. Nayeemul Islam Nayeem et al. [14] applied the YOLOv11 architecture, typically used for object detection, to behavior recognition tasks. Through standardized preprocessing of 512 × 512 pixel images and data augmentation techniques such as flipping and rotation, they effectively enhanced the model’s generalization ability for 19 types of dynamic and static behaviors. Their adaptive learning rate optimization strategy further broke through the accuracy bottleneck in multi-class behavior recognition. In the domain of sensor data processing, Yasin Kaya et al. [15] proposed a 1D-CNN model, demonstrating strong feature extraction capabilities by directly learning from raw accelerometer and gyroscope data. This model achieved a 98% accuracy rate in recognizing 12 behavior classes in the UCI-HAPT dataset through parameter optimization, offering an efficient solution for health monitoring and abnormal behavior detection in wearable device contexts. Mamta Ghalan et al. [16] combined GCN with CNNs to create an integrated model. Leveraging GCN’s ability to model the topological relationships of skeletal joint movements, this model effectively addressed the challenge of recognizing nonlinear complex behaviors in the UniMiB-SHAR dataset and overcame the limitations of traditional CNNs in capturing spatiotemporal joint features. This work provides an engineering reference for target-level feature extraction in violence detection.

### 2.2. Research Status of Violence Behavior Detection Based on Deep Learning

Early violent behavior video detection often relied on processing indicators such as blood, flames, and explosions, with violent scenes typically derived from movies. The earliest detection methods were proposed by Nam et al. [17] in 1998, who evaluated video clips by detecting flames in explosions and gunshots, as well as blood and sounds in violent scenes. Clarin et al. [18] identified a representative frame within a scene and labeled skin, blood, and non-skin, non-blood pixels using the Self-Organizing Map algorithm. These labeled pixels were then grouped using Connected Components to identify areas where violent behavior might have occurred. Subsequently, the pixel change ratio map (PCRM) for these regions was cross-checked, and the motion intensity of each pixel in all frames of the scene was determined. If the PCRM found that pixels in the identified skin and blood components had high motion intensity, the scene was considered to contain violent behavior.

With the exceptional performance of deep learning in image classification, speech recognition, object detection, and other tasks, the adoption of deep learning methods for detecting violent behavior has become a natural choice. Sudhakaran et al. [19] used CNNs to extract frame-level features from videos and then employed LSTM variants to aggregate these features, using the differences between adjacent frames as input to force the model to encode changes occurring in the video. Ravanbakhsh et al. [20] employed Generative Adversarial Networks (GANs) to detect anomalies in videos. Since the training process only involved normal data, the model was unable to learn abnormal data characteristics. During testing, motion features reconstructed by the GAN were compared with input motion features, and local differences were computed to detect anomalies and their respective regions. Roy et al. [21] proposed a two-stage object-centered adversarial framework. The first stage learned the correspondence between the current appearance of objects in normal scenes and past gradient images, allowing the generation of past gradients from the current appearance. The second stage extracted parts from real and generated images with normal object behavior to reconstruct errors, using adversarial training to detect abnormal violent behaviors through the learned differences from normal data. This approach provided a novel solution for scenarios with sparse data.

Researchers have also conducted extensive work on technical optimization. Wang Jingwen [22] focused on lightweight solutions and proposed a YOLOv8-based lightweight violent detection algorithm, which performed well on the RWF-2000 dataset with low computational complexity. Liu Lin’en [23] introduced a deep and shallow feature fusion network that extracts optical flow and grayscale frame difference features to construct motion-combined images, balancing real-time performance and accuracy through multi-scale feature extraction, channel attention, and ConvLSTM. They also incorporated the Video MAE framework into violent behavior detection, retaining the encoder and adding multi-layer perceptrons and classifiers, along with Vision Transformer self-supervised pre-training and fine-tuning. This approach demonstrated excellent performance on the RLVS and RWF2000 datasets. To address technical challenges, Liu Lin’en utilized a pre-trained model as the backbone network in the deep and shallow feature fusion network, enhancing sample information with motion-combined images and alleviating the small sample problem. Chen Hao [24] proposed a multi-level gated dual-stream network, capturing deep temporal features with LSTM and keyframe shallow features with multi-head attention blocks. The network combined gated connection modules for early fusion of spatio-temporal flow features, effectively solving the problem of confusing behavior differentiation, and outperforming traditional methods on multiple datasets. They also introduced a multi-modal attention multi-stream network, incorporating a speech stream to extract speech features, combined with a visual stream for appearance and motion features. The three-way attention fusion addressed the issue of visual feature loss caused by occlusion. Yan Baozhong [25] improved YOLOv4, optimizing the backbone network and post-processing methods. After training on a self-built dataset, they achieved 98% mAP and 56.4 fps detection speed, meeting real-time monitoring requirements. Wang Yanjie et al. [26] reviewed deep learning-based multi-person abnormal behavior detection methods, clarifying concepts and classifications, analyzing feature extraction and detection methods, and comparing dataset and model performance, thereby providing theoretical guidance for future research.

### 2.3. Weakly Supervised Video Anomaly Detection

In recent years, weakly supervised video anomaly detection has gradually become a research hotspot due to its advantage of reducing annotation costs in practical applications. Hu Ying [27] proposed an Att-BLSTM-based method that integrates attention mechanisms with bidirectional LSTM to achieve temporal modeling of anomalous segments under video-level labels. Zhao Yizheng [28] introduced a self-training algorithm based on label confidence, which iteratively improves pseudo-label quality and effectively alleviates the noise interference inherent in weakly supervised settings. Tang Jun, Zhang Yin, and Wang Ke [29] proposed a feature-difference learning approach that enhances discriminability by constructing contrasts between normal and abnormal features. Zhang Yifan, Yan Yu, and Liu Teli [30] developed a cross-dimensional interactive framework driven by a three-dimensional rearranged MLP, enabling efficient fusion of multi-dimensional features and precise localization of abnormal segments. In addition, Qu Yi [31] systematically investigated the mechanisms of weakly supervised anomaly detection, further extending related application scenarios.

At the international level, Karim, Doshi, and Yilmaz [32] proposed a real-time weakly supervised video anomaly detection method, which significantly improved inference efficiency while maintaining detection accuracy. Wu, Zhou, and Pang et al. [33] introduced a spatio-temporal prompt-based detection framework in ACM Multimedia, which strengthens the saliency of anomalous regions by guiding feature learning. Pu, Wu, and Yang et al. [34] proposed a Prompt-Enhanced Context Feature learning approach that leverages prompt mechanisms to enhance contextual feature representation. Su, Tan, and An et al. [35] designed a semantic-driven dual consistency learning framework, which effectively improves generalization ability under weak supervision through cross-semantic and cross-temporal consistency constraints. These studies demonstrate that prompt learning, context modeling, and consistency constraints have become emerging trends in weakly supervised video anomaly detection.

Furthermore, to address the challenge of anomaly localization under weak supervision, Ullah and Khan et al. [36] proposed detection methods based on sequential attention mechanisms. By explicitly modeling long-range dependencies along the temporal dimension, these methods improved segment-level localization accuracy. Overall, both domestic and international research has converged on multiple technical pathways for weakly supervised video anomaly detection: (i) methods focusing on label noise and pseudo-label quality control, such as self-training and consistency learning; (ii) approaches emphasizing spatio-temporal modeling and prompt mechanisms, enhancing feature representations for more accurate anomaly discrimination; and (iii) efforts toward efficient inference and practical deployment, facilitating the application of algorithms in public safety and surveillance. These advances not only improve detection performance under weak supervision but also lay a solid theoretical and methodological foundation for building more intelligent video surveillance systems in the future.

Nevertheless, existing methods still face challenges, such as insufficient adaptability to complex scenarios and inadequate multi-modal information fusion. Therefore, this paper focuses on enhancing the robustness, adequacy of feature representation, and adaptability of weakly supervised approaches in complex monitoring environments. By leveraging the advantages of multi-scale feature fusion and multi-modal learning, this study aims to explore efficient feature extraction and fusion strategies within a weakly supervised framework. The goal is to reduce the dependence on annotated data while improving model performance in challenging monitoring scenarios, such as sudden lighting changes and occlusions, ultimately providing an optimal solution for engineering-oriented violence detection.

As summarized in Table 1, a comparison of representative weakly supervised methods highlights their supervision type, core techniques, main contributions, and limitations.

## 3. Methods

**Figure 1:**
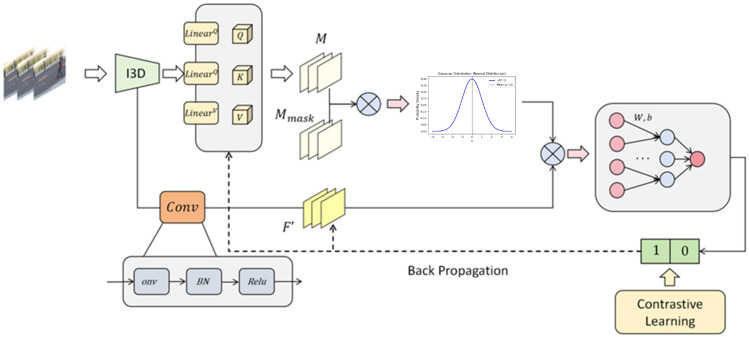
Model architecture. The framework first reduces and encodes the input feature sequence. A temporal structural attention mechanism with causal masking and temporal decay weights is then applied to capture both short- and long-term dependencies. Finally, contrastive learning with positive and negative offsets enlarges the separation between abnormal and normal segments in the latent space, and the model is trained end-to-end under the overall loss function.

This section provides a detailed description of the proposed model, including the temporal structural attention mechanism, the contrastive learning strategy, and the loss function design. The overall workflow is as follows: first, the input feature sequence is reduced in dimension and preliminarily encoded; then, temporal attention is introduced, incorporating a causal mask and temporal decay weights to enhance the modeling of temporal dependencies; finally, contrastive learning is employed, where positive and negative offsets are used to enlarge the distributional gap between abnormal and normal segments in the latent space, and the model is trained in an end-to-end manner under the guidance of the overall loss function. The overall architecture of the model is illustrated in Figure 1.

### 3.1. Temporal Structural Attention Mechanism

To better model the global temporal correlations of video features, we adopt the idea of self-attention in the temporal dimension and incorporate a causal mask together with temporal decay to design an attention module tailored for anomaly detection. The input video is divided into multiple consecutive segments, and a pretrained convolutional neural network is adopted as the feature extractor to obtain the initial feature of each segment, which are then assembled into an initial feature matrix. A convolution operation is subsequently applied to the initial feature matrix for dimensionality reduction, yielding the first video feature representation. Specifically, the input video refers to the raw material to be subjected to anomaly detection, which may come from real-time monitoring footage in intelligent surveillance scenarios or from historical recordings in security analysis. The video is segmented into *T* consecutive and non-overlapping clips at fixed time intervals. The segmentation length can be set according to the frame rate and task requirements to ensure coverage of the complete temporal information. A convolutional neural network pretrained on large-scale video datasets (e.g., the Kinetics dataset), such as the Inflated 3D ConvNet (I3D ConvNet), is then employed to extract semantic features for each clip. Let the initial feature of each segment be denoted as $f_{t}\in R^{D}$, and the features of all segments are combined into a matrix $F\in R^{T\times D}$. Before entering the attention module, a 1×1 convolution is applied to reduce the dimensionality, yielding the compact representation $F^{\prime }\in R^{T\times d}$:

(1) $$F^{\prime }=Conv_{1\times 1}(F)$$

where *F*′ denotes the first video feature, *F* is the initial feature matrix, *D* is the dimension of the initial features and d<D is typically set to a smaller value to reduce computational cost.

Subsequently, to construct the self-attention structure, three linear mappings (implemented in this work using fully connected layers) are introduced to generate the query, key, and value representations, denoted as Q,K and V:

(2) $$Q={\mathrm{Linear}}^{Q}(F^{\prime }),K={\mathrm{Linear}}^{K}(F^{\prime }),V={\mathrm{Linear}}^{V}(F^{\prime })$$

In the temporal dimension, we first compute the dot-product similarity among all segments to obtain the attention map M:

(3) $$M=QK^{T}\in R^{T\times T}$$

Subsequently, to enforce temporal order constraints on the attention matrix, we define a causal mask matrix $M_{mask}\in {\left\{0,1\right\}}^{T\times T}$, which ensures that the current position *i* can only attend to itself and preceding temporal segments, while the influence of future frames is masked out:

(4) $$M_{mask}(i,j)=\left\{\begin{array}{c} 1,\;\mathrm{if}\;j\leq i\\ 0,\;\mathrm{if}\;j>i \end{array}\right.$$

**Figure 2:**
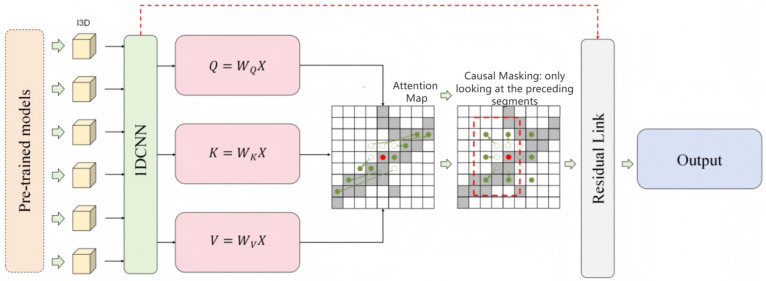
Architecture of the proposed temporal structural attention module, where causal masking ensures that only preceding segments are considered to prevent information leakage. Gray cells: represent all positions in the full attention matrix, i.e., where attention links can be formed between any two time segments (in essence, each cell corresponds to a query–key pair). White circles: indicate the positions that are actually attendable under the causal constraint—namely, the attention links from the current segment to the allowed set of historical segments. Diagonal line: denotes strong correlation along the diagonal. Red dot: indicates the target segment currently being processed for attention computation. Green dots: indicate highly relevant (high-correlation) segments.

The causal attention matrix is then obtained:

(5) $$M^{causal}=M⊗M_{mask}$$

where ⊗ denotes element-wise multiplication (Hadamard product). During both training and inference, all entries corresponding to future segments are set to zero or significantly suppressed, thereby retaining only the attention information from the current and past segments. In this way, the model does not “see” future frames during training or inference, which prevents information leakage and better aligns with the real temporal order, thus improving the modeling of sequential dependencies in practical scenarios. Its module architecture is shown in Figure 2.

Furthermore, considering that the importance of different temporal distances varies for the current segment, a temporal decay weight $W_{time}\in R^{T\times T}$ is introduced into the attention mechanism, which is typically defined in the form of exponential or Gaussian decay:

(6) $$W_{time}(i,j)=\exp(-\alpha |i-j|)$$

where α>0 is a hyperparameter that controls the degree of decay as the temporal distance increases. This decay factor is then incorporated into the attention map:

(7) $$M^{td}=M_{causal}⊗W_{time}$$

Based on Equation (7), a normalization operation is applied to map the numerical values into the range [0,1]:

(8) $$M^{\prime }=\mathrm{softmax}(M^{td})$$

Finally, the attention output incorporating the temporal dimension is obtained:

(9) $$F_{att}^{\prime }=M^{\prime }V$$

The attention output $F_{att}^{\prime }$ and the original feature $F^{\prime }$ are combined through residual addition or concatenation to obtain the final feature representation X final for subsequent processing, as follows:

(10) $$X=F^{\prime }+F_{att}^{\prime }$$

Intuitively, the causal mask ensures that each video segment only attends to its own history, similar to how humans rely on past experiences without foreseeing the future. Meanwhile, the temporal decay weight reflects a natural “forgetting effect,” where the influence of more distant segments gradually weakens. Together, these mechanisms allow the model to emphasize temporally relevant patterns while suppressing noise from distant or irrelevant information.

Through the aforementioned design, the temporal structure attention module can explicitly model both short-term and long-term dependencies along the temporal dimension. Specifically, the causal mask constrains the direction of attention propagation, ensuring that the current timestep attends only to itself and its historical segments, without accessing future frames. This design aligns with the true causal nature of video events. Such a constraint effectively enhances the model’s responsiveness to short-term local dynamics, such as sudden movements, object displacements, or transient abnormal behaviors.

In addition, the temporal decay weights adaptively adjust the attention strength based on temporal distance, assigning higher weights to nearby segments while exponentially decaying the influence of distant ones. This mechanism avoids excessive smoothing while preserving essential long-term contextual dependencies, enabling the model to track the evolution of abnormal events over extended temporal spans. For instance, when an anomalous behavior persists or gradually escalates, the temporal decay component helps the model remain sensitive to its continuity and progression.

### 3.2. Architecture of the Contrastive Learning Module

**Figure 3:**
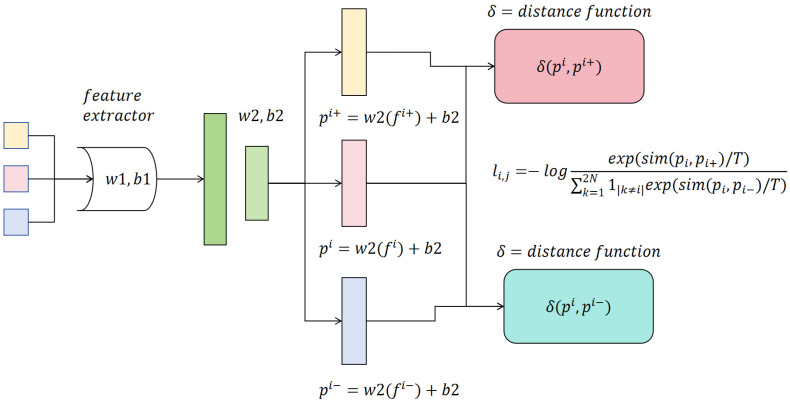
Architecture of the Contrastive Learning Module. The process begins with feature extraction, followed by two linear projections to obtain the embeddings. Positive samples and negative samples are constructed with respect to an anchor. We use a distance function δ(·,·) defined as the Euclidean distance, δ(u, v) = ∥u − v∥_2_. The contrastive loss in this figure follows the InfoNCE formulation described in Equation (15).

To further enhance the discrimination of abnormal segments in the latent space, we introduce the concept of contrastive learning, leveraging positive and negative offsets to amplify the differences between abnormal and normal segments. The architecture of the contrastive learning module is illustrated in the following Figure 3:

As illustrated in Figure 3, the diagram presents the feature representation method based on contrastive learning. After feature extraction through the feature extractor, linear transformations are applied to map the features into the embedding space, where the similarity between positive and negative samples is computed to optimize the InfoNCE loss function. The objective is to maximize the similarity between positive pairs while minimizing that between negative pairs, thereby enhancing the discriminative capability for anomaly detection. Specifically, an offset $A_{t}$ is predicted using $F^{\prime }$ described in Section 3.1, which is regarded as the output of a lightweight network *Net*:

(11) $$A_{t}=Net({F_{t}}^{\prime })$$

Here, ${F_{t}}^{\prime }$ denotes the feature of the *t* segment. If the *t* segment is a normal segment, we apply a “positive offset” in the feature space, i.e.,

(12) $$F_{t}^{normal}={F^{\prime }}_{t}+A_{t}$$

For abnormal segments, a “negative offset” is applied:

(13) $$F_{t}^{abnormal}={F^{\prime }}_{t}-A_{t}$$

In weakly supervised settings, it is impossible to directly determine whether a specific segment is abnormal. Therefore, we employ video-level labels combined with a contrastive learning approach, aiming to automatically approximate the latent distribution of “normal/abnormal” offsets during training. Given a batch of videos $\left\{X_{i}\right\}$ with labels $y_{i}\in \{0,1\}$, the contrastive relationship is defined as follows: if $y_{i}=1$, then some segments should be pushed farther away from normal segments in the offset space; if $y_{i}=0$ (normal video), all segments should remain clustered with normal segments after the offset.

We then introduce the following distance measure between the offset features:

(14) $$sim(u,v)=\frac{u^{T}v}{\left||u||||v|\right|}$$

Suppose the “positive sample” corresponding to a certain feature F (after offset) is denoted as $F^{+}$, while its set of “negative samples” is denoted as $\left\{F_{1}^{-},F_{2}^{-},\ldots ,F_{N}^{-}\right\}$. Within the contrastive learning framework, we define a core contrastive objective: the distance in the feature space between abnormal segments after offset and normal segments after offset should be maximized, thereby enhancing their separability. At the same time, the distance between normal segments should be minimized so that they remain tightly clustered in the latent space, ensuring stable learning of normal patterns.

This objective allows the model to effectively separate abnormal and normal features, making abnormal segments more distinguishable in the latent space and thus improving anomaly detection performance under weakly supervised scenarios. To achieve this goal, we adopt the InfoNCE loss (InfoLoss), which enforces constraints by maximizing the similarity of positive pairs and minimizing the similarity of negative pairs. In this way, the model can automatically learn more stable representational differences between abnormal and normal segments, thereby improving detection accuracy and robustness. The corresponding formula is given as follows:

(15) $$L_{\mathrm{infoNCE}}=-\log\frac{\exp(sim(F,F^{+})/\tau )}{\sum_{j=1}^{N}\exp(sim(F,F_{j}^{-})/\tau )+\exp(sim(F,F^{+})/\tau )}$$

Here, τ>0 is the temperature hyperparameter used to adjust the sensitivity of the logits. Through Equation (15), the model can continuously strengthen the similarity between abnormal segments and abnormal segments during training, while pushing abnormal segments farther away from normal segments in the feature space, thereby improving the discriminative ability for anomaly detection. In addition, the InfoNCE loss maximizes the consistency of features among samples from the same class while minimizing the similarity between samples from different classes, essentially establishing a relation-based learning mechanism in the latent space. This relative discrimination property makes it particularly suitable for weakly supervised tasks: even without precise segment-level annotations, the model can leverage video-level labels and inter-sample similarities to adaptively pull closer the distributions of samples from the same class and push apart those from different classes, thereby obtaining more discriminative feature representations. In practical implementation, positive and negative sample pairs are determined based on the labels, and in this study, we traverse all video segments $x_{t}$ to construct the corresponding positive and negative pairs, which are then used to form the batch-wise loss:

(16) $$L_{\mathrm{info}}=\frac{1}{\left|\Omega \right|}\sum_{x\in \Omega }L_{\mathrm{infoNce}}(F)$$

Here, Ω includes all segments available for contrastive learning in the current training batch, and $L_{\mathrm{infoNce}}(F)$ is computed according to Equation (15). Furthermore, to reduce the noise introduced by weak supervision, we apply a weighting to the selected positive pairs, where the weights are based on the “confidence” scores provided by video-level labels. The weight $w(x_{t})$ reflects the confidence of whether the segment is recognized as abnormal or normal. Accordingly, the contrastive loss can be expressed as:

(17) $$L_{\mathrm{info}}=\frac{1}{\sum_{x\in \Omega }w(x)}\sum_{x\in \Omega }w(x)L_{\mathrm{infoNce}}(F)$$

In this way, the training process can focus more on reliable pairs while mitigating the influence of irrelevant noise. Finally, to further stabilize the offset effect, we additionally impose a distance-separating constraint between the abnormal center $c_{a}$ and the normal center $c_{n}$, similar to some contrastive learning methods, i.e.,

(18) $$L_{center}=\max(0,m-||c_{a}-c_{n}||)$$

where $c_{a}=\frac{1}{\Omega_{a}}\sum_{x\in \Omega_{a}}F$, $c_{n}=\frac{1}{\Omega_{n}}\sum_{x\in \Omega_{n}}F$ represent the abnormal and normal centers of the offset features, respectively. When $\left||c_{a}-c_{n}|\right|$ becomes sufficiently large, the loss will no longer increase, so as to prevent excessive separation from affecting the learning of other components.

Finally, the above losses are combined to form the final contrastive loss:

(19) $$L_{\mathrm{contrastive}}=\alpha L_{\mathrm{info}}+\beta L_{\mathrm{center}}$$

Here, α and β are tunable weights. By minimizing $L_{\mathrm{contrastive}}$, the model can make abnormal offset features and normal offset features clearly separable in the latent space, thereby overcoming the difficulty of directly judging the authenticity of segments under weak supervision. Meanwhile, by incorporating the strategy of positive and negative offsets, abnormal segments in training are guided to move toward the “abnormal” direction, while normal segments remain relatively compact, thus enhancing both the accuracy and robustness of anomaly detection.

Furthermore, we present the final overall loss, in which an additional cross-entropy loss is incorporated to further improve classification performance. The overall loss function is formulated as

(20) $$L_{all}=L_{cross-entropy}+L_{\mathrm{contrastive}}$$

where $L_{cross-entropy}$ uses the features from Equation (10), and its computation is defined as follows:

(21) $$L_{cross-entropy}=-\frac{1}{N}\sum_{i=1}^{N}\left[y_{i}\log{\hat{y}}_{i}(X_{i})+(1-y_{i})\log(1-{\hat{y}}_{i}(X_{i}))\right]$$

Here, N denotes the number of samples and C denotes the number of classes. This formula can further enhance the classification capability of distinguishing anomalies. The overall algorithmic workflow is illustrated in Algorithm 1.

**Algorithm 1:** Overall pseudocode of the proposed algorithm**Require:** Video segment features $F=\{f_{t}{\}}_{t=1}^{T},$ labels $y_{i}\in \{0,1\}$, hyperparameters $\tau ,\alpha ,m,\alpha_{w},\beta$

**Ensure:** Total loss $L_{all}$ 
1:

$F^{\prime }\leftarrow {Conv}_{1\times 1}\left(F\right)$

2:

$Q,K,V\leftarrow Linear\left(F^{\prime }\right)$

3:$M\leftarrow QK^{T}$                                                                                                     ▷ causal mask4:for i=1 to T do 5:        **for** j=1 to T do 6:                **if** j>i then7:                        $M_{ij}\leftarrow 0$8:                **end if**9:                $M_{ij}\leftarrow M_{ij}\cdot exp\left(-\alpha \left|i-j\right|\right)$                                             ▷ temporal distance decay10:        **end for**11:
**end for**
12:

$M^{\prime }\leftarrow softmax\left(M\right)$

13:

$F_{att}\leftarrow M^{\prime }V$

14:

$X\leftarrow {F^{\prime }+F}_{att}$

15:**for** each segment *t* **do**16:        $A_{t}\leftarrow Net\left(F_{t}^{\prime }\right)$17:        $F_{t}^{normal}\leftarrow F_{t}^{\prime }+A$18:        $F_{t}^{abnormal}\leftarrow F_{t}^{\prime }-A$19:
**end for**
20:**for** each segment $x_{t}$ **do**21:        Construct positive and negative sample pairs and compute $L_{\mathrm{infoNCE}}\left(x_{t}\right)$22:
**end for**
23:

$L_{info}\leftarrow \sum w\left(x_{t}\right)L_{\mathrm{infoNCE}}\left(x_{t}\right)$ $L_{\mathrm{info}}=\frac{1}{\sum_{x\in \Omega }w(x)}\sum_{x\in \Omega }w(x)L_{\mathrm{infoNce}}(F)$

24:Compute class centers $\;c_{a},c_{n}$ and obtain $L_{\mathrm{center}}=\max\left(0,m-\left|\left|c_{a}-c_{n}\right|\right|\right)$25:

$L_{\mathrm{contrastive}}\leftarrow \alpha L_{info}+\beta L_{\mathrm{center}}$

26:
**Cross-entropy loss:**
         $L_{\mathrm{cross}\text{-}\mathrm{entropy}}=-\frac{1}{N}\sum_{i=1}^{N}\left[y_{i}\log\hat{y}\left(X_{i}\right)+\left(1-y_{i}\right)\log\left(1-\hat{y}\left(X_{i}\right)\right)\right]$
27:
**Total loss:**
      $L_{\mathrm{all}}\leftarrow L_{\mathrm{cross}\text{-}\mathrm{entropy}}+L_{\mathrm{contrastive}}$
28:**return** $L_{\mathrm{all}}$


As shown in Figure 3, in the proposed framework, Net(⋅) is a lightweight multilayer perceptron designed to perform a nonlinear mapping on the segment features output by the temporal structural attention module, thereby predicting the latent offset $A_{t}$ for each segment. This offset is subsequently used to construct positive and negative sample pairs, enabling explicit feature-level discrimination.

### 3.3. Datasets and Evaluation Metrics

#### 3.3.1. Dataset Overview

1. ShanghaiTech Dataset

The ShanghaiTech dataset is a widely used benchmark for video anomaly detection research, primarily collected from multiple street surveillance scenes in Shanghai. It covers 13 different scenarios and contains a total of 437 videos, including 307 normal videos and 130 abnormal videos. The abnormal events are diverse, such as pedestrians intruding into motor vehicle lanes, vehicles violating traffic regulations, and crowd gatherings. These abnormal behaviors typically occur in public places, providing high practical value for real-world applications. In addition, the relatively low proportion of anomalies makes the detection task even more challenging.

**Figure 4:**
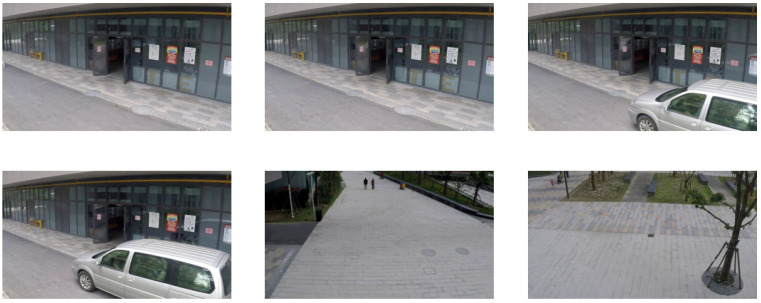
Consecutive video frame examples from the ShanghaiTech dataset, illustrating subtle variations between adjacent frames in normal traffic scenes, which highlight the challenge of temporal dynamics in anomaly detection.

In its original setting, the ShanghaiTech dataset was mainly used for unsupervised anomaly detection, where the model could only access normal videos during training and was required to detect abnormal frames during testing. However, to meet the needs of weakly supervised learning research, subsequent studies reorganized the dataset to include a portion of abnormal videos for training. Under this weakly supervised setting, only video-level normal/abnormal labels are provided for training data, without frame-level annotations. This setting is more consistent with real-world application scenarios, enabling anomaly detection methods to be more practical in surveillance tasks. An example of the dataset is illustrated in Figure 4.

2. UCF-Crime Dataset

The UCF-Crime dataset, developed by the University of CentralFlorida, is a video dataset dedicated to anomaly detection, particularly suitable for the automatic recognition of violent behaviors and criminal activities. It contains approximately 128 h of surveillance video footage, comprising 1900 untrimmed real-world scene videos. The dataset covers 13 categories of abnormal behaviors, including violent events such as abuse, fighting, and robbery, as well as other anomalous activities such as burglary and arson. In addition, a large number of normal behavior segments are included for comparison.

**Figure 5:**
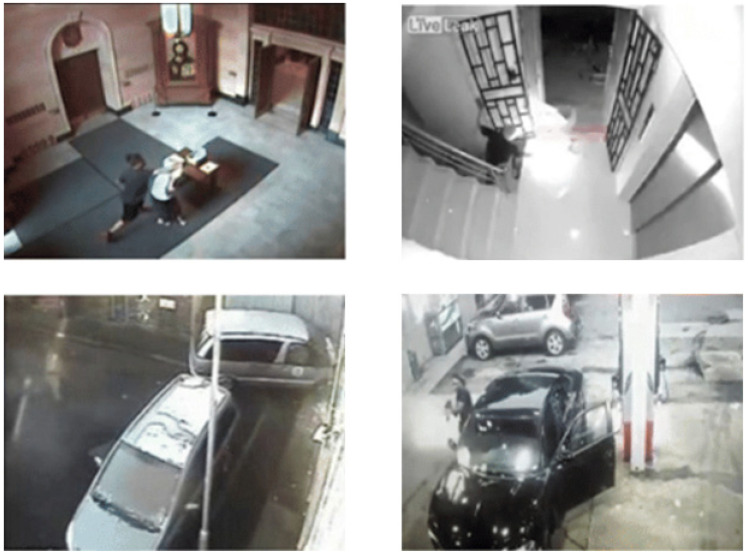
Examples from the UCF-Crime dataset. The dataset contains untrimmed real-world surveillance videos covering diverse abnormal behaviors such as abuse, fighting, robbery, burglary, and arson, as well as normal activities for comparison. These examples illustrate the complexity and variability of scenarios in public safety monitoring, which make anomaly detection tasks more realistic and challenging.

The primary motivation for designing this dataset was to advance research in video analysis technology, especially in the domain of public safety, by providing resources to train models capable of detecting abnormal behaviors in real-world scenarios. The videos exhibit long temporal continuity and are annotated with precise timestamps of abnormal events, making the dataset an important resource for research in action recognition and anomaly detection. It has been widely adopted in the fields of deep learning and computer vision. An example of the UCF-Crime dataset is illustrated in Figure 5.

#### 3.3.2. Evaluation Metrics Overview

In video anomaly detection tasks, commonly used evaluation metrics include Accuracy, F1-score, and Area Under the Curve (AUC). These metrics are employed to assess the performance of models in classification and anomaly detection tasks, providing an effective evaluation of their detection capability and robustness.

1. ACC

Accuracy (ACC) is the most fundamental classification evaluation metric, representing the proportion of correctly classified samples to the total number of samples. Its calculation formula is as follows:

(22) $$ACC=\frac{TP+TN}{TP+TN+FP+FN}$$

where TP denotes the abnormal segments correctly detected as abnormal,TN denotes the normal segments correctly detected as normal, FP denotes the normal segments misclassified as abnormal, and FN denotes the abnormal segments missed. When the data distribution across categories is balanced, accuracy can effectively reflect the classification capability of the model. However, in anomaly detection tasks, since abnormal samples are usually much fewer than normal ones, relying solely on ACC may lead to bias.

2. F1-Score

The F1-score is a comprehensive metric used to balance precision and recall, and is particularly suitable for tasks with imbalanced class distributions, such as anomaly detection. Its calculation formula is

$$F1=2\times \frac{\mathrm{Precision}\times \mathrm{Recall}}{\mathrm{Precision}+\mathrm{Recall}}$$

Here, Precision represents the proportion of true abnormal segments among those detected as abnormal, while Recall represents the proportion of abnormal segments correctly detected out of all abnormal segments. Compared with Accuracy, the F1-score provides greater reference value in anomaly detection tasks, as it simultaneously takes into account both false positives and false negatives.

3. AUC

AUC refers to the Area Under the ROC Curve, which is used to measure the classification capability of a model under different threshold settings. The ROC curve is plotted with the False Positive Rate (FPR) on the x-axis and the True Positive Rate (TPR) on the y-axis. The calculation formulas are

$$TPR=\frac{TP}{TP+FN},\;FPR=\frac{FP}{FP+TN}$$

The value of AUC ranges from 0 to 1, with higher values indicating better model performance. Compared with ACC and F1-score, AUC is more suitable for anomaly detection tasks with relatively large variations in decision thresholds, as it effectively evaluates the overall detection capability of a model across different operating points. Therefore, in weakly supervised video anomaly detection tasks, AUC is a commonly used evaluation metric.

## 4. Results

In this section, we conduct a comprehensive experimental evaluation of the proposed method, including comparative experiments with existing approaches, hyperparameter sensitivity analysis, ablation studies, and visualization-based analyses. First, in the comparative experiments, we compare our method with several mainstream anomaly detection models to validate its performance advantages across different datasets. Second, in the hyperparameter sensitivity experiments, we analyze the impact of key hyperparameters (such as the temperature parameter and contrastive learning weight) on model performance, in order to assess its stability and robustness. Subsequently, we carry out ablation studies by gradually removing or replacing critical modules of the model to verify the contribution of each component to the overall performance. Finally, in the visualization experiments, we present feature distribution visualizations, attention heatmaps, and anomaly segment detection results to intuitively demonstrate the effectiveness and interpretability of the proposed model in anomaly detection tasks.

### 4.1. Experimental Setup

The experiments were conducted in a high-performance computing environment, primarily utilizing an NVIDIA RTX 4090D (24 GB) GPU (NVIDIA, Santa Clara, CA, USA) for training to accelerate the processing of large-scale video data. Each computing node was equipped with a 15-core Intel Xeon Platinum 8474C processor (Intel, Santa Clara, CA, USA), 80 GB of memory, and 1 TB of SSD storage. All experiments were implemented based on the PyTorch 2.5.1 framework and accelerated with CUDA 12.1 to enhance training efficiency.

For hyperparameter settings, we adopted SGD as the optimizer, with an initial learning rate of 0.001, a momentum parameter of 0.9, and a weight decay of 0.0005 to improve optimization stability. For contrastive loss, the temperature parameter was set to τ = 0.07, and the loss weights were set to α = 0.5 and β = 0.5. Furthermore, in the weakly supervised scenario, video-level labels were employed for training. The batch size was set to 32, and the maximum number of training epochs was set to 200, with an early stopping strategy to prevent overfitting. In addition, a cosine annealing learning rate decay strategy was applied to further enhance training efficiency. The detailed experimental settings are summarized in Table 2.

### 4.2. Results of Comparative Experiments

In this subsection, we present the results of the comparative experiments. However, due to the significant differences between the ShanghaiTech and UCF-Crime datasets in terms of scene scale and feature extraction protocols, this study adopts dataset-specific mainstream baselines in the comparative experiments to ensure fairness and comparability of the results. For the ShanghaiTech dataset, most existing methods (e.g., Conv-AE, RTFM) employ frame-level RGB features as input; therefore, we follow this convention for a consistent comparison. In contrast, for the UCF-Crime dataset, the community standard predominantly utilizes I3D-RGB or optical flow features for feature extraction (e.g., BODS, RTFM, GODS). To align with prior studies, we uniformly adopt I3D-RGB features as input for evaluation on this dataset.

First, the experimental results on the ShangHaiTech dataset are reported, as shown in Table 3.

From the experimental results, it can be observed that our proposed method achieves the best performance on the ShangHaiTech dataset, surpassing existing approaches in terms of AUC, ACC, and F1-Score, with values reaching 98.11%, 96.13%, and 94.52%, respectively. Compared with the previous state-of-the-art method RTFM, our model demonstrates improvements across all key metrics, indicating that the proposed temporal structure attention mechanism combined with the contrastive learning strategy effectively enhances the model’s discriminative capability in anomaly detection tasks. In particular, for the AUC metric, our method achieves a 0.9% improvement over RTFM, further reducing the distributional overlap between abnormal and normal segments, thereby rendering anomaly detection under weakly supervised scenarios more stable and accurate.

Analyzing the performance of different models, it is evident that traditional autoencoder-based methods exhibit inferior performance in terms of AUC, with values of only 60.85% and 71.20%, respectively. This suggests that relying solely on reconstruction error for anomaly detection is insufficient to capture complex temporal structures. Meanwhile, GCN-Anomaly, which leverages graph neural networks, achieves relatively good AUC results, but still falls far short compared with RTFM and our method, highlighting the critical importance of temporal modeling in video anomaly detection. Furthermore, methods such as VEC and MNAD, which incorporate memory modules, obtain certain improvements in ACC and F1-Score but remain less competitive compared to RTFM and our approach. Overall, our method consistently outperforms existing baselines across multiple evaluation metrics, fully demonstrating its effectiveness and robustness.

Subsequently, we report the experimental results on the UCF-Crime dataset, as summarized in Table 4.

The experimental results demonstrate that our proposed method achieves an AUC score of 84.47% on the UCF-Crime dataset, surpassing the previous state-of-the-art method RTFM and achieving leading performance on this benchmark. Compared with traditional approaches such as SVM-Base, deep learning–based anomaly detection methods exhibit significant advantages, indicating that relying solely on simple statistical features or reconstruction errors is insufficient for effectively capturing anomalous patterns. While BODS and GODS achieve certain improvements by leveraging I3D RGB features, considerable room for optimization remains, suggesting that local feature modeling alone is inadequate for precise anomaly detection. Moreover, the Motion-Aware method, which is based on optical flow features, further validates the importance of motion information in anomaly detection. However, compared with GCN-Anomaly, which utilizes TSN RGB features, its global modeling capability is still limited.

In terms of high-performance approaches, GCN-Anomaly introduces graph neural networks to model temporal relationships for the first time and achieves clear improvements over methods that rely solely on local features. Nevertheless, both RTFM and our method exploit I3D RGB features and integrate temporal attention mechanisms with contrastive learning strategies, thereby further enhancing the discriminative power of the model in identifying anomalous segments. Notably, our method achieves a 0.17% improvement in AUC over RTFM. Although the gain appears modest, it nonetheless demonstrates that the proposed temporal structure modeling and contrastive learning strategies can effectively strengthen the robustness of anomaly detection, enabling the model to more stably detect anomalous events under weakly supervised scenarios.

### 4.3. Experimental Results of Hyperparameter Sensitivity

Furthermore, this paper presents the experimental results of hyperparameter sensitivity analysis. We first report the basic hyperparameter sensitivity results, including the fundamental hyperparameters learning rate (Lr) and optimizer. The results of the optimizer experiments are shown in Table 5.

From the experimental results, it can be observed that different optimizers exhibit varying performance on the ShangHaiTech dataset, among which SGD achieves the best AUC, ACC, and F1-Score. This indicates that SGD demonstrates superior convergence and generalization ability in anomaly detection tasks. In contrast, Adam and AdamW also achieve comparable performance, with AUC values of 97.92% and 97.85%, respectively. However, their ACC and F1-Score are slightly lower than those of SGD. This suggests that although the Adam family of optimizers generally performs well in deep learning tasks, their adaptive learning rate may reduce the model’s sensitivity to subtle differences in anomalous patterns, thereby affecting the final detection accuracy. Moreover, AdaGrad exhibits the weakest performance, with an AUC of only 95.78%, implying that its cumulative gradient update strategy may reduce the learning rate in the later stages of training, thus limiting the model’s ability to learn anomalous features.

Overall, Adam and AdamW benefit from their adaptivity, enabling faster convergence and strong performance during the early stages of training. However, compared to SGD, their final generalization ability is slightly inferior. By contrast, SGD, owing to its stable gradient updates and strong momentum acceleration, is better suited to capturing complex temporal feature learning, thereby achieving the best performance in weakly supervised anomaly detection tasks. Furthermore, the experimental results of the initial learning rate are presented in Table 6.

From the experimental results, it can be observed that the choice of learning rate has a significant impact on model performance. When the learning rate is relatively large (e.g., 0.01), the AUC drops to 96.45%, and both ACC and F1-Score are relatively low. This may be due to the large step size causing instability during training, potentially leading to missed optimal solutions. As the learning rate decreases, the model performance improves. Learning rates of 0.002 and 0.003 achieve results close to optimal, but are still slightly inferior to the setting of 0.001. Ultimately, a learning rate of 0.001 achieves the best AUC (98.11%), ACC (96.13%), and F1-Score (94.52%), indicating that a smaller learning rate enables more stable convergence of the model.

In addition to the basic hyperparameter sensitivity experiments, this study also fine-tuned the weighting of the loss function in Equation (19). The corresponding experimental results are presented in Table 7.

The experimental results indicate that different combinations of α and β have a significant impact on model performance. When α = 0.5 and β = 0.5, the model achieved the best performance, with an AUC of 98.11%, ACC of 96.13%, and F1-Score of 94.52%, suggesting that this parameter setting provides the optimal balance between the loss terms. When α was relatively large, although the F1-Score remained high, the AUC decreased compared to the optimal setting, indicating a decline in anomaly detection capability. Conversely, when both α and β were too small, the overall performance dropped significantly, which may be attributed to the model’s inability to sufficiently optimize the contrastive loss for abnormal segments.

### 4.4. Ablation Study Results

Subsequently, the results of the ablation study are presented on two different datasets. First, the results on the ShangHaiTech dataset are reported, as shown in Table 8.

The experimental results demonstrate that each newly added module contributes to enhancing the model’s performance. The baseline model achieved an AUC of 97.21%, an ACC of 94.85%, and an F1-Score of 92.67%, indicating that the basic approach already possesses strong anomaly detection capability. With the addition of temporal structural attention, the AUC increased to 97.68%, and both ACC and F1-Score also improved, suggesting that incorporating temporal dimension modeling can effectively enhance the detection of anomalous behaviors. Furthermore, after introducing the contrastive loss, the AUC reached 97.85%, which indicates that contrastive learning strengthens the discriminability between anomalous and normal segments, thereby improving detection performance. Finally, our method (Ours) achieved the best performance across AUC, ACC, and F1-Score, validating the effectiveness of temporal structural attention and contrastive loss, whose combined effect significantly enhances anomaly detection capability. Subsequently, the ablation results on the UCF-Crime dataset are presented in Table 9.

From the experimental results, our method demonstrates improved performance on the UCF-Crime dataset through the introduction of temporal structural attention and contrastive loss. The baseline model achieved an AUC of 84.30%. When temporal structural attention was added, the AUC slightly decreased to 84.22%, which may be attributed to the varying dependency on temporal information in anomaly detection tasks, where temporal modeling could sometimes introduce noise. However, after incorporating contrastive loss, the AUC increased to 84.35%, indicating that this loss effectively enhances the discriminability between abnormal and normal segments. Finally, by combining both enhancements, our method (ours) achieved the best AUC of 84.47%, surpassing the baseline model and the results of adding either module individually. This demonstrates that the synergistic effect of temporal structural attention and contrastive loss further optimizes the model’s discriminative ability and enhances overall detection performance.

### 4.5. Visualization of Experimental Results

**Figure 6:**
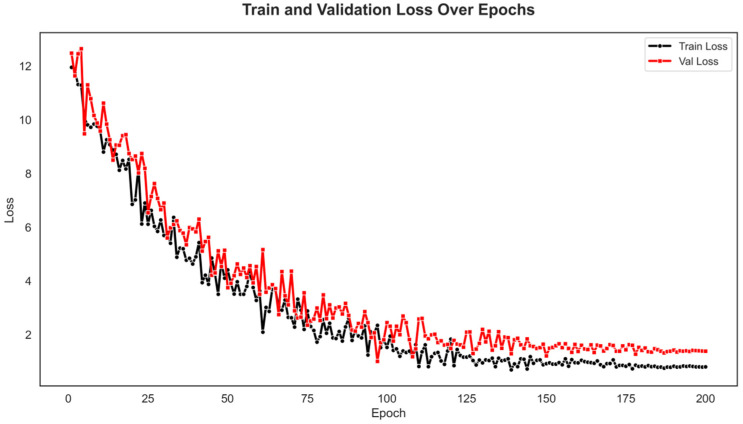
Loss curves during the training process on the ShangHaiTech dataset. The figure illustrates the convergence trends of both training loss (black curve) and validation loss (red curve) over 200 epochs. As shown, the overall losses exhibit a clear downward trend with fluctuations gradually stabilizing, indicating that the proposed model achieves effective optimization and maintains strong generalization ability throughout the training process.

Finally, this paper presents the visualization results of the experiments. First, the loss function curve during the training process is provided, as shown in Figure 3. Finally, this paper presents the visualization results of the experiments. First, the loss function curve during the training process is provided, as shown in Figure 6.

**Figure 7:**
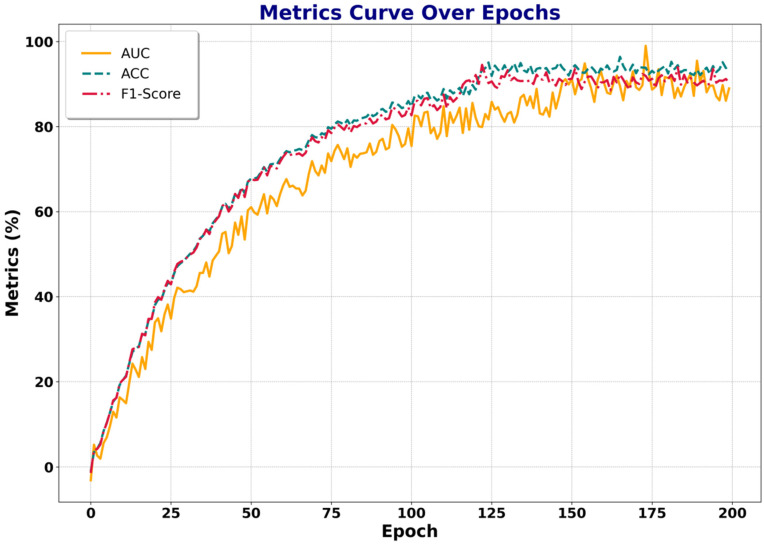
Variations in evaluation metrics over epochs (ShangHaiTech). This figure illustrates the changes in AUC, ACC, and F1-Score during the training process on the ShangHaiTech dataset. As the number of epochs increases, all three metrics steadily improve and eventually converge, indicating the effectiveness and stability of the proposed method in achieving optimal anomaly detection performance.

Subsequently, the variations in different evaluation metrics with respect to epochs are presented, as shown in Figure 7.

From the loss curve, it can be observed that both the training loss (Train Loss) and validation loss (Val Loss) gradually decrease as the number of epochs increases and approach stability near 200 epochs. This indicates that the model continuously learns more effective features during training and successfully converges. The decreasing trends of training and validation losses are generally consistent, and the final gap between them remains small, suggesting that the model achieves balanced performance on both the training and validation sets without obvious overfitting. The slightly larger fluctuations in the validation loss may result from the imbalance of abnormal samples and the noise introduced by the weakly supervised setting.

From the metric curves, AUC, ACC, and F1-Score all gradually increase with the number of training epochs and stabilize around 150 epochs. Among them, ACC and F1-Score exhibit smoother growth and remain stable after approximately 100 epochs, showing that the model has effectively learned to distinguish normal from abnormal segments with strong discriminative ability. AUC grows rapidly in the early stages but still exhibits certain fluctuations after 150 epochs. This may be attributed to the feature learning of abnormal segments, where the model’s recognition ability for specific abnormal patterns fluctuates at certain stages. Nonetheless, the overall trend remains upward, eventually achieving high detection performance.

In summary, the model not only demonstrates effective loss reduction during training but also achieves continuous improvement in the three core metrics—AUC, ACC, and F1-Score—ultimately converging to an optimal performance level. These results confirm that the proposed temporal structural attention mechanism and contrastive loss function can significantly enhance anomaly detection capability, enabling the model to more accurately distinguish between normal and abnormal behaviors, thereby exhibiting strong generalization and robustness in weakly supervised video anomaly detection tasks.

**Figure 8:**
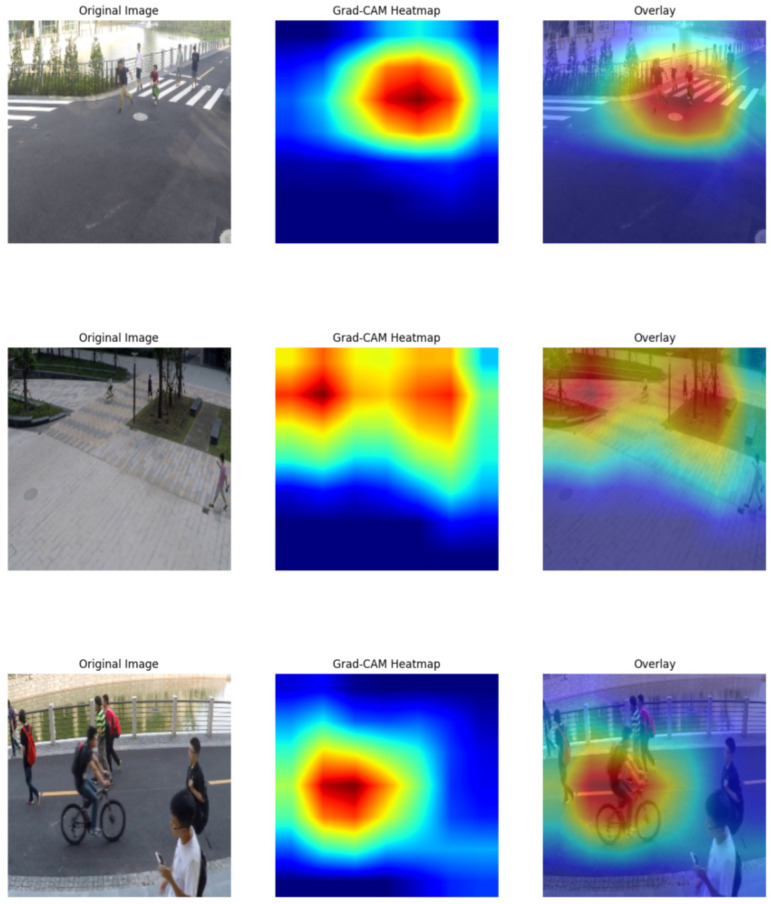
Grad-CAM heatmap visualization results (ShangHaiTech). The figure shows Grad-CAM heatmaps on ShangHaiTech samples, where the highlighted regions indicate the model’s focus when detecting abnormal events.

Next, the Grad-CAM visualization results are presented, where the ShangHaiTech dataset is selected as the test set. The corresponding results are shown in Figure 8.

From the visualization results of the Grad-CAM heatmaps, it can be observed that the model is able to accurately focus on key regions when detecting abnormal behaviors and successfully identify different types of abnormal events. In the first-row example, the abnormal behavior in the original image is a fight, and the high-response area of the heatmap is concentrated at the location where the fight occurs. This indicates that the model can correctly focus on the abnormal crowd rather than the background or irrelevant areas. This suggests that the model is capable of recognizing not only individual behaviors but also abnormal patterns in multi-person interactions. In the second and third rows, the abnormal behavior is bicycle riding. As shown in the heatmaps, the model’s attention accurately covers the riders rather than the surrounding pedestrians or background. This demonstrates the model’s ability to effectively distinguish between normal walking behavior and riding behavior, further validating its accuracy in abnormal behavior detection.

**Figure 9:**
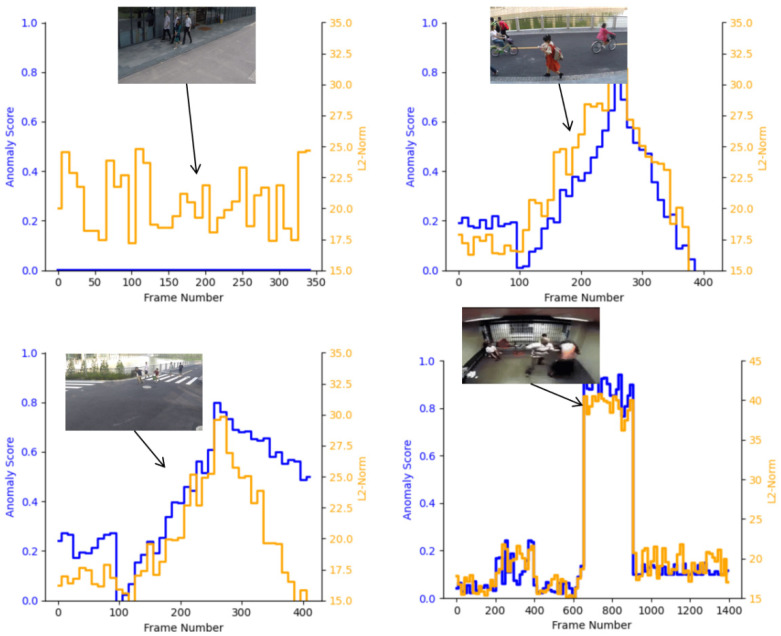
Comparison between anomaly scores and feature magnitudes on the ShangHaiTech and UCF-Crime datasets. The blue curves denote anomaly scores and the orange curves denote feature magnitudes, with sample frames illustrating corresponding abnormal events.

Regarding the color distribution of the heatmaps, the red regions represent the areas most likely to contain abnormal events, while the blue regions correspond to areas of low attention. It can be observed that the model consistently localizes abnormal events in different scenarios, such as the fight in the first row and the cyclists in the second and third rows. This shows that the proposed temporal-structural attention mechanism can effectively model temporal abnormal behaviors, while the contrastive loss further enhances the feature discriminability between abnormal and normal behaviors. Moreover, the coverage of the heatmaps is highly consistent with the actual occurrence of abnormal events, confirming the effectiveness of the proposed method. Consequently, the model achieves not only high accuracy in abnormal detection but also strong interpretability. Finally, the anomaly scores and feature magnitudes on both the UCF-Crime and ShangHaiTech datasets are presented, as shown in Figure 9.

From the experimental results, our method demonstrates effective anomaly detection on both the UCF-Crime and ShangHaiTech datasets, with a strong correlation observed between anomaly scores and feature magnitudes. Specifically, during the intervals when abnormal events occur, both the anomaly score and L2-Norm show a significant increase, indicating that the model can accurately capture anomalous behaviors and assign high-confidence predictions to abnormal segments. For example, in cases such as bicycle riding and fighting, both the blue and orange curves exhibit sharp fluctuations at the time of anomaly, reflecting the model’s sensitivity in detecting abnormal behaviors. In contrast, during normal segments, the anomaly scores and L2-Norm remain at relatively low levels, confirming that the model avoids excessive false alarms and maintains detection stability and robustness.

Across different scenarios, the model responds effectively to both short-term sudden anomalies and long-term persistent anomalies. Notably, even in complex indoor environments, the anomaly detection scores remain at high levels, suggesting strong generalization ability. In some cases, the increase in anomaly scores shows a slight delay, which may be related to the frame-level feature extraction and temporal modeling process. This limitation could be addressed in future work by incorporating more advanced temporal attention mechanisms to further enhance the model’s response speed. Overall, these results validate the effectiveness of the proposed method, showing that it achieves not only high accuracy but also strong interpretability and robustness in anomaly detection.

### 4.6. Analysis of Failure Cases

**Figure 10:**
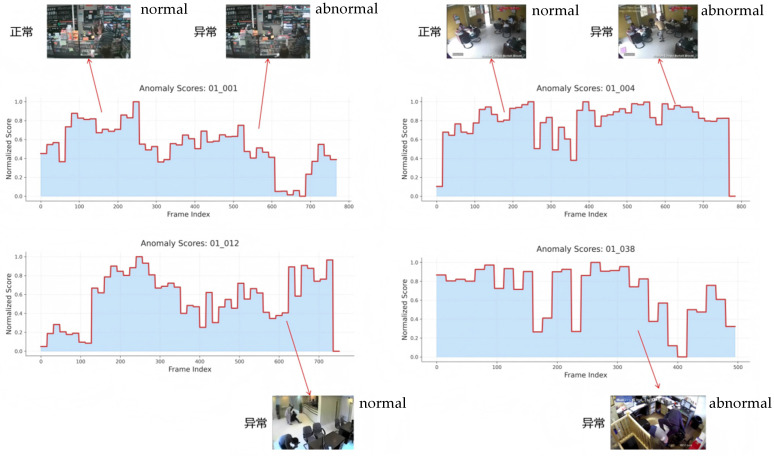
Failure case analysis of the proposed method. The anomaly scores of selected video segments are illustrated, where the model shows instability in complex scenarios (e.g., illumination changes, crowded scenes, or subtle anomalies), resulting in fluctuations, delayed responses, or misdetections.

Furthermore, this paper also conducts a failure case analysis, and the experimental results are presented in Figure 10.

As shown in Figure 10, the model exhibits instability in anomaly detection for a small number of video segments, manifested as large fluctuations in anomaly scores or delayed responses to certain true abnormal events. These failure cases primarily occur in scenes with complex backgrounds, significant illumination changes, or high crowd density, where the model struggles to distinguish normal dynamic variations from actual abnormal behaviors, resulting in false alarms or missed detections. In some videos, anomalies of very short duration or with subtle visual changes also cause the anomaly scores to fail to rise in time, indicating that the model still has certain limitations in fine-grained temporal localization. Overall, these failure cases reveal the potential weaknesses of the model under extreme conditions and highlight the insufficiency of current open-source datasets in providing adequate samples for complex scenarios such as anomaly sparsity.

### 4.7. System Efficiency and IoT-Oriented Resource Evaluation

To verify the suitability of the proposed method for real-world IoT-based intelligent surveillance deployments, this section evaluates the computational complexity, model size, and system-level overhead. Modern smart-city video surveillance systems typically consist of AI-enabled cameras equipped with embedded GPU/NPU accelerators and standard cameras connected to edge video servers responsible for decoding, storage, and multi-stream management. Therefore, it is essential that the anomaly detection module introduces only marginal additional resource consumption so that it can be integrated into existing pipelines without exceeding hardware constraints.

#### 4.7.1. Computational Capability and Configuration of IoT Devices

In practical deployments, both AI-enabled IP cameras (e.g., dome cameras, bullet cameras, face-capture devices) and standard cameras are typically equipped with embedded deep-learning accelerators or connected to edge video servers capable of running multiple task. According to mainstream commercial specifications of smart cameras and video management equipment, the front-end IoT devices typically provide:

- Embedded GPU/NPU with 2–8 TOPS of compute capability;
- 1–4 GB on-board memory;
- Real-time processing requirements of 10–25 FPS per video stream;
- Edge video servers providing multi-core CPUs and additional GPU/ASIC acceleration.

Under this hardware profile, any additional module for anomaly detection must remain compact and computationally lightweight to operate alongside other analytics tasks.

#### 4.7.2. Model Complexity and Computational Cost

The proposed framework introduces two lightweight components on top of the pre-extracted I3D features:

- A temporal structural attention module;
- A contrastive learning head.

The temporal structural attention module introduces only lightweight matrix multiplications with causal masking, while the contrastive learning predictor consists of two small fully connected layers. No heavy computation is used in our framework. The CNN backbone (I3D) is not part of the deployed module because many AI cameras and standard cameras already provide feature extraction capabilities through built-in deep-learning accelerators or edge processing units. Therefore, only the newly added components contribute to the system overhead.

The complexity of the proposed metric model for anomaly detection heads is shown in Table 10.

These numbers indicate that the proposed modules are significantly lighter than typical on-camera models for face recognition or pedestrian detection, which commonly exceed 5–20 million parameters.

#### 4.7.3. Runtime and System Overhead Evaluation

To further evaluate the practicality of the proposed model in real-world systems, this section tests and estimates the inference speed and system-level overhead of the anomaly detection head.

In our experimental environment, the measured inference time of the anomaly detection head is approximately: approximately 0.35 milliseconds per clip, corresponding to an inference speed of around 2800 frames per second.

This result indicates that, compared with the feature extraction backbone, the computational overhead introduced by the anomaly detection head itself is almost negligible. When this module is migrated to embedded GPU/NPU platforms (e.g., devices with 2–8 TOPS of compute capability), a proportional estimation based on compute capacity suggests that the additional load generally accounts for no more than 3% of the total compute budget, while still supporting 15–20 FPS real-time processing for a single video stream.

In terms of memory, the peak additional usage of the anomaly detection head is less than 200 MB, which leaves a substantial margin compared with the 1–4 GB on-board memory commonly available on current AI-enabled cameras and edge servers.

In summary, the proposed anomaly detection framework achieves competitive performance on benchmark datasets such as ShanghaiTech and UCF-Crime while introducing only minimal computational and storage overhead. The experimental results demonstrate that the method offers good resource adaptability and engineering deployability for IoT devices in smart-city video surveillance scenarios.

## 5. Conclusions and Future Work

This paper systematically evaluated the effectiveness of the proposed temporal structural attention mechanism and contrastive learning loss for video anomaly detection. We first presented the model architecture in detail, highlighting how the temporal structural attention mechanism enhances the ability to model sequential dependencies and how the contrastive learning loss optimizes the feature distribution between abnormal and normal segments, thereby improving the discriminative capability of the model. Subsequently, comprehensive experiments were conducted on the ShangHaiTech and UCF-Crime benchmark datasets, including comparative studies, hyperparameter sensitivity analysis, ablation studies, and visualization experiments. The results demonstrated that our method consistently outperforms existing state-of-the-art approaches across key evaluation metrics such as AUC, ACC, and F1-Score, further validating its robustness and generalization capability.

In addition, visualization experiments—including loss curves, performance trends, and Grad-CAM heatmaps—intuitively illustrate the convergence process and anomaly detection ability of the model. The results indicate that the model not only distinguishes abnormal from normal segments with high accuracy but also effectively captures the temporal occurrence of abnormal events. Moreover, the strong correlation observed between anomaly scores and feature magnitudes in anomalous regions further substantiates the effectiveness of the proposed approach in modeling temporal dynamics and feature-level contrast.

In summary, the proposed temporal structural attention mechanism and contrastive learning loss demonstrate superior performance in weakly supervised video anomaly detection tasks, achieving improvements in both detection accuracy and robustness while enhancing model interpretability.

Looking ahead, this framework still offers considerable room for extension and optimization in future research and practical applications. On one hand, further lightweight design is necessary to balance accuracy with computational efficiency, enabling deployment in real-time and resource-constrained scenarios such as edge devices and mobile anomaly detection systems. Model compression and distillation strategies can be explored to reduce inference latency while maintaining robust performance in large-scale video streams. On the other hand, transfer learning and domain adaptation represent promising directions to enhance the practical value of our approach by allowing the model to adapt to the complexity and diversity of real-world surveillance data, thus improving cross-domain generalization and reducing retraining costs. Moreover, incorporating multimodal information (e.g., audio and sensor data) and advanced spatio-temporal modeling can further improve the robustness of anomaly recognition under complex environments. Finally, uncertainty modeling and more comprehensive evaluation protocols will help quantify prediction reliability and provide stronger support for downstream decision-making. Overall, this study not only verifies the superiority of the proposed method on benchmark datasets but also lays a solid foundation for the long-term development and application of video anomaly detection.

## 6. Patents

This work has resulted in a patent titled “A video anomaly detection method, device, equipment, and storage medium” (Patent Application No. 2025113462806), applied on 19 September 2025 [49]. The patent is related to the methods/technologies discussed in this manuscript.

## Figures and Tables

**Table 1:** Comparison of representative weakly supervised video anomaly detection methods in recent years.

Author and Year	Supervision Type	Core Technique/Method	Main Contribution	Limitation/Drawback
Hu Ying et al. (2021) [27]	Weak supervision	Att-BLSTM	Combined attention mechanism with Bi-LSTM for video-level anomaly modeling	Insufficient modeling of long-term dependencies
Zhao Yizheng et al. (2022) [28]	Weak supervision	Label confidence self-training	Iterative pseudo-label refinement to mitigate noise interference	High dependence on initial pseudo-labels
Tang Jun et al. (2022) [29]	Weak supervision	Feature-difference learning	Enhanced discriminability by contrasting normal and abnormal features	Limited ability in modeling spatio-temporal changes in complex scenes
Zhang Yifan et al. (2023) [30]	Weak supervision	3D-rearranged MLP	Achieved cross-dimensional feature interaction and efficient anomaly localization	Limited ability in modeling spatio-temporal changes in complex scenes
Wu et al. (2024) [33]	Weak supervision	Spatio-temporal prompting	Guided the model to focus on salient anomalous regions	Prompt design is experience-dependent, poor transferability
Su et al. (2024) [35]	Weak supervision	Semantic consistency learning	Cross-semantic and cross-temporal consistency improved generalization	Relatively high computational complexity
Ullah et al. (2025) [36]	Weak supervision	Sequential attention	Modeled long-range dependencies to improve segment-level localization	Limited robustness across different scenarios

**Table 2:** Experimental settings: detailed hardware, software, and training configurations adopted for model implementation.

Setting	Detailed Configuration
Hardware Environment	NVIDIARTX4090D(24 GB)GPU
	IntelXeonPlatinum8474C(15 cores)
	80 GB Memory
	1 TGB System Disk
Software Environment	PyTorch + CUDA
Optimizer	SGD (momentum = 0.9, weight decay = 0.0005)
Initial Learning Rate	0.001
Learning Rate Scheduler	CosineAnnealing
Temperature Parameter (τ)	0.07
Contrastive Loss Wights	0.5, 0.5
Batch Size	32
Training Epochs	200

**Table 3:** Quantitative comparison results on the ShangHaiTech dataset in terms of AUC, ACC, and F1-Score. The best results are highlighted in bold, and the second-best results are underlined.

Model	AUC	ACC	F1-Score
Conv-AE [37]	60.85	-	-
Stack-RNN [38]	68.00	-	-
Frame-Pred [39]	73.40	-	-
Mem-AE [40]	71.20	-	-
MNAD [41]	70.50	68.42	66.13
VEC [42]	74.80	71.20	-
GCN-Anomaly [43]	84.44	79.31	76.75
AR-NET [44]	91.24	-	-
RTFM [45]	97.21	94.85	92.67
Ours	**98.11**	**96.13**	**94.52**

**Table 4:** Quantitative comparison results on the UCF-Crime dataset in terms of AUC and Feature. The best results are highlighted in bold, and the second-best results are underlined.

Model	Feature	AUC
SVM-Base [46]	-	50.00
Conv-AE	-	50.60
BODS	I3D RGB	68.26
GODS [47]	I3D RGB	70.46
Motion-Aware [48]	PWCFlow	79.00
GCN-Anomaly	TSN RGB	82.12
RTFM	I3D RGB	84.30
Ours	I3D RGB	**84.47**

**Table 5:** Quantitative results of different optimizers on the ShangHaiTech dataset, where AUC, ACC, and F1-Score are reported for comparison.

Optimizer	AUC	ACC	F1-Score
AdaGrad	95.78	93.42	91.13
AdamW	97.85	95.67	93.89
Adam	97.92	95.78	94.10
SGD	98.11	96.13	94.52

**Table 6:** Quantitative results of initial learning rate (Lr) experiments on the ShangHaiTech dataset, where AUC, ACC, and F1-Score are reported for different Lr values.

Lr	AUC	ACC	F1-Score
0.01	96.45	94.32	92.10
0.003	97.68	95.42	93.75
0.002	97.92	95.78	94.10
0.001	98.11	96.13	94.52

**Table 7:** Experimental results of parameter settings for α and β on the ShangHaiTech dataset.

Parameter Settings	AUC	ACC	F1-Score
α = 0.1, β = 0.1	95.34	92.85	90.72
α = 0.3, β = 0.7	96.78	94.32	92.05
α = 0.7, β = 0.3	97.65	95.20	93.12
α = 0.5, β = 0.5	98.11	96.13	94.52

**Table 8:** Ablation study results on the ShangHaiTech dataset. The table reports the performance of the baseline model and the incremental addition of temporal structural attention and contrastive loss function.

Model	AUC	ACC	F1-Score
Baseline Model	97.21	94.85	92.67
+Temporal Structural Attention	97.68	95.42	93.25
+Contrastive Loss Function	97.85	95.78	93.89
Ours	98.11	96.13	94.52

**Table 9:** Ablation study results on UCF-Crime, where each additional module is evaluated to demonstrate its contribution. The results show that our method achieves the highest AUC, validating the effectiveness of combining temporal structural attention with contrastive loss.

Model	AUC
Baseline Model	84.30
+Temporal Structural Attention	84.22
+Contrastive Loss Function	84.35
Ours	84.47

**Table 10:** Model complexity and resource usage for the anomaly detection head.

Metric	Value
Parameters (temporal attention + contrastive head)	0.72 M
Model size	≈2.9 MB
FLOPs per video (T = 32 segments)	≈1.1 GFLOPs
Peak memory usage	<200 MB

## Data Availability

The data presented in this study are available on request from the corresponding author.

## References

[1] Sultani W., Chen C., Shah M. Real-world anomaly detection in surveillance videos. Proceedings of the IEEE Conference on Computer Vision and Pattern Recognition.

[2] Liu Y., Liu S., Zhu X., Yang H., Li J., Guo J., Teng L., Yang D., Wang Y., Liu J. (2025). Privacy-preserving video anomaly detection: A survey. IEEE Trans. Neural Netw. Learn. Syst..

[3] Elmetwally A., Eldeeb R., Elmougy S. (2025). Deep learning based anomaly detection in real-time video. Multimed. Tools Appl..

[4] Lloyd K., Marshall D., Moore S.C., Rosin P.L. (2017). Detecting violent and abnormal crowd activity using temporal analysis of grey level co-occurrence matrix (GLCM) based texture measures. Mach. Vis. Appl..

[5] Wang Y., Gao Z., Long M., Wang J., Yu P.S. (2018). PredRNN++: Towards A Resolution of the Deep-in-Time Dilemma in Spatiotemporal Predictive Learning. arXiv.

[6] Song G., Qian Y., Wang Y. (2025). STGCN-PAD: A Spatial-Temporal Graph Convolutional Network for Detecting Abnormal Pedestrian Motion Patterns at Grade Crossings. Pattern Anal. Appl..

[7] Hu H., Liu Y., Wang J., Zhang Z. Noise-Resistant Video Anomaly Detection via RGB Error-Guided Multiscale Predictive Coding. Proceedings of the IEEE/CVF Conference on Computer Vision and Pattern Recognition (CVPR).

[8] Zhan C., Wang H., Hu H. (2025). Human Action Recognition Algorithm Based on Deformable 3D Convolution and Bert Temporal Modeling. J. Changchun Univ. Technol..

[9] Zhang H., Feng J. (2024). A Review of Human Action Recognition Based on Deep Learning Methods. J. Jiyuan Vocat. Tech. Coll..

[10] Gong Y., Yang W., Jin K. (2024). Pedestrian Abnormal Behavior Detection Based on Deep Learning. China New Technol. Prod..

[11] Guo J., Ye J., Kong Y., Chen J., He G., Yao Z., Ye S., Peng Y., Liu S., Feng D. (2024). Research Progress of Human Action Recognition Based on Deep Learning. J. Zhongkai Univ. Agric. Eng..

[12] Feng L. (2023). Human Action Recognition Method Based on Convolutional Neural Network. J. Tonghua Norm. Univ..

[13] Talukder A., Kazi M., Uddin A. (2025). HARNet: An enhanced deep learning-based human activity recognition model. Clust. Comput..

[14] Nayeem N.I., Mahbuba S., Disha S.I., Buiyan M.R.H., Rahman S., Abdullah-Al-Wadud M., Uddin J. (2025). A YOLOv11-Based Deep Learning Framework for Multi-Class Human Action Recognition. Comput. Mater. Contin..

[15] Kaya Y., Topuz E.K. (2024). Human activity recognition from multiple sensors data using deep CNNs. Multimed. Tools Appl..

[16] Ghalan M., Aggarwal R.K. (2024). Novel Human Activity Recognition by graph engineered ensemble deep learning model. IFAC J. Syst. Control.

[17] Nam J., Alghoniemy M., Tewfik A.H. Audio-visual content-based violent scene characterization. Proceedings of the 1998 International Conference on Image Processing.

[18] Clarin C., Dionisio J., Echavez M., Naval P. (2005). DOVE: Detection of movie violence using motion intensity analysis on skin and blood. PCSC.

[19] Sudhakaran S., Lanz O. Learning to detect violent videos using convolutional long short-term memory. Proceedings of the 2017 14th IEEE International Conference on Advanced Video and Signal Based Surveillance.

[20] Ravanbakhsh M., Nabi M., Sangineto E., Marcenaro L., Regazzoni C., Sebe N. Abnormal event detection in videos using generative adversarial nets. Proceedings of the 2017 IEEE International Conference on Image Processing.

[21] Roy P.R., Bilodeau G.A., Seoud L. Local Anomaly Detection in Videos using Object-Centric Adversarial Learning. Proceedings of the International Conference on Pattern Recognition.

[22] Wang J. (2024). Research on Lightweight Violent Behavior Detection Algorithm Based on Deep Learning. Ph.D. Thesis.

[23] Liu L. (2025). Research on Violent Behavior Detection Based on Deep Spatiotemporal Feature Modeling. Ph.D. Thesis.

[24] Chen H. (2024). Research on Violent Behavior Detection Based on Deep Learning. Ph.D. Thesis.

[25] Yan B., Liu Z. (2022). Real-time Violent Behavior Detection Algorithm Based on YOLOv4. Appl. Sci. Technol..

[26] Wang Y., Wang X., Zhao L., Zhuang X. (2024). A Review of Research on Multi-person Abnormal Behavior Detection Based on Deep Learning. J. Front. Comput. Sci. Technol..

[27] Hu Y. (2025). Research on Weakly Supervised Video Anomaly Detection Based on Att-BLSTM. Master’s Thesis.

[28] Zhao Y. (2025). Weakly Supervised Self-Training Video Anomaly Detection Algorithm Based on Label Confidence. High Technol. Commun..

[29] Tang J., Zhang Y., Wang K., Bao W. (2025). Weakly Supervised Video Anomaly Detection Algorithm Based on Feature Difference Learning. J. Huazhong Univ. Sci. Technol. (Nat. Sci. Ed.).

[30] Zhang Y., Yan Y., Liu T., Chen P. (2025). Cross-Dimensional Interactive Weakly Supervised Video Anomaly Detection Driven by 3D Rearranged MLP. J. Front. Comput. Sci. Technol. (Comput. Sci. Explor.).

[31] Qu Y. (2025). Research on Weakly Supervised Video Anomaly Event Detection Algorithms. Ph.D. Thesis.

[32] Karim H., Doshi K., Yilmaz Y. Real-Time Weakly Supervised Video Anomaly Detection. Proceedings of the IEEE/CVF Winter Conference on Applications of Computer Vision (WACV).

[33] Wu P., Zhou X., Pang G., Yang Z., Yan Q., Wang P., Zhang Y. Weakly Supervised Video Anomaly Detection and Localization with Spatio-Temporal Prompts. Proceedings of the 32nd ACM International Conference on Multimedia (ACM MM).

[34] Pu Y., Wu X., Yang L., Wang S. (2024). Learning Prompt-Enhanced Context Features for Weakly Supervised Video Anomaly Detection. IEEE Trans. Image Process..

[35] Su Y., Tan Y., An S., Xing M., Feng Z. (2025). Semantic-Driven Dual Consistency Learning for Weakly Supervised Video Anomaly Detection. Pattern Recognit..

[36] Ullah W., Ullah F.U.M., Khan Z.A., Baik S.W. (2023). Sequential Attention Mechanism for Weakly Supervised Video Anomaly Detection. Expert Syst. Appl..

[37] Hasan M., Choi J., Neumann J., Davis L.S. Learning Temporal Regularity in Video Sequences. Proceedings of the IEEE Conference on Computer Vision and Pattern Recognition (CVPR).

[38] Luo W., Liu W., Gao S. A Revisit of Sparse Coding Based Anomaly Detection in Stacked RNN Framework. Proceedings of the IEEE International Conference on Computer Vision (ICCV).

[39] Liu W., Luo W., Lian D., Gao S. Future Frame Prediction for Anomaly Detection—A New Baseline. Proceedings of the IEEE Conference on Computer Vision and Pattern Recognition (CVPR).

[40] Gong D., Liu L., Le V., van den Hengel A., Shen C., Reid I., Shi Q. Memorizing Normality to Detect Anomaly: Memory-Augmented Deep Autoencoder for Unsupervised Anomaly Detection. Proceedings of the IEEE/CVF International Conference on Computer Vision (ICCV).

[41] Park H., Noh J., Ham B. Learning Memory-Guided Normality for Anomaly Detection. Proceedings of the IEEE/CVF Conference on Computer Vision and Pattern Recognition (CVPR).

[42] Yu G., Wang S., Cai Z., Yu M., Lin W., Ren H., Li S. Cloze Test Helps: Effective Video Anomaly Detection via Learning to Complete Video Events. Proceedings of the 28th ACM International Conference on Multimedia (ACM MM).

[43] Cao C., Zhang X., Zhang S., Wang H., He R. (2022). Adaptive Graph Convolutional Networks for Weakly Supervised Anomaly Detection in Videos. IEEE Signal Process. Lett..

[44] Wan B., Fang Y., Xia X., Bai S., Li Z. (2021). Weakly Supervised Video Anomaly Detection via Center-Guided Discriminative Learning. arXiv.

[45] Tian Y., Pang G., Chen Y., Singh R., Verjans J., Carneiro G. Weakly-Supervised Video Anomaly Detection with Robust Temporal Feature Magnitude Learning. Proceedings of the IEEE/CVF International Conference on Computer Vision (ICCV).

[46] Wang X. (2024). Support Vector Machine-Based Video Anomaly Detection Approaches. Anomaly Detection in Video Surveillance.

[47] Wang J., Cherian A. GODS: Generalized One-Class Discriminative Subspaces for Anomaly Detection. Proceedings of the IEEE/CVF International Conference on Computer Vision (ICCV).

[48] Zhu Y., Newsam S. (2019). Motion-Aware Feature for Improved Video Anomaly Detection. arXiv.

[49] Beijing University of Posts and Telecommunications (2025). Video Anomaly Detection Method, Apparatus, Device and Storage Medium. Chinese Patent Application.

